# Accuracy of transvaginal sonography versus magnetic resonance imaging in the diagnosis of rectosigmoid endometriosis: Systematic review and meta-analysis

**DOI:** 10.1371/journal.pone.0214842

**Published:** 2019-04-09

**Authors:** Ana Paula Carvalhal Moura, Helizabet Salomão Abdalla Ayroza Ribeiro, Wanderley Marques Bernardo, Ricardo Simões, Ulysses S. Torres, Giuseppe D’Ippolito, Marc Bazot, Paulo Augusto Ayrosa Galvão Ribeiro

**Affiliations:** 1 Grupo Fleury, Sao Paulo, Brazil; 2 Santa Casa de Misericórdia de Sao Paulo, Sao Paulo, Brazil; 3 Universidade Federal de Sao Paulo, Sao Paulo, Brazil; 4 Department of Radiology, Tenon Hospital, Assistance Publique des Hôpitaux de Paris, Université Pierre et Marie Curie Paris, Paris, France; University of Vermont Larner College of Medicine, UNITED STATES

## Abstract

**Introduction:**

Intestinal endometriosis is considered the most severe form of deep endometriosis, the rectosigmoid being involved in about 90% of cases of bowel infiltration. Transvaginal sonography (TVS) and magnetic resonance imaging (MRI) have been used for noninvasive diagnosis and preoperative mapping of rectosigmoid endometriosis (RE), but no consensus has been reached so far regarding which method is the most accurate in this setting.

**Objective:**

We aimed at performing a systematic review and meta-analysis to compare the accuracy of TVS versus MRI in the diagnosis of RE in a same population.

**Methods:**

A systematic review was conducted in accordance with the PRISMA guidelines. Studies were identified by searching the MEDLINE, Embase, and LILACS databases, as well the reference lists of retrieved articles, through February 2019. We included all cross-sectional studies that evaluated the accuracy of TVS versus MRI in the diagnosis of RE within a same sample of subjects and that used surgical findings with histological confirmation as the gold standard. The QUADAS-2 instrument was used to evaluate study quality. Sensitivity, specificity, positive likelihood ratios (LR+), and negative likelihood ratios (LR-) for the diagnosis of RE were calculated. This study is registered with PROSPERO, number CRD42017064378.

**Results:**

Eight studies (*n* = 1132) were included in the meta-analysis. The pooled sensitivity, specificity, LR+, and LR- values of MRI for RE were 90% (95% CI, 87–92%), 96% (95% CI, 94–97%), 17.26 (95% CI, 3.57–83.50), and 0.15 (95% CI, 0.10–0.23); values of TVS were 90% [95% CI, 87–92%], 96% (95% CI, 94–97%), 20.66 (95% CI, 8.71–49.00) and 0.12 (95% CI, 0.08–0.20), respectively. Areas under the S-ROC curves (AUC) showed no statistically significant differences between MRI (AUC = 0.948) and TVS (AUC = 0.930) in the diagnosis of RE (*P* = 0.13). Moreover, considering the average prevalence among the studies of 47.3%, both methods demonstrated similarly high positive post-test probabilities (93.9% for TVS and 94.8% for MRI), and the combined use of them yielded a post-test probability of 99.6%.

**Conclusion:**

MRI and TVS have similarly high accuracy and positive post-test probabilities in the noninvasive diagnosis of RE. Combination of MRI and TVS may increase even further the positive post-test probabilities to near 100%.

## Introduction

Endometriosis is defined as the presence of endometrial-like tissue (glands and/or stroma) outside the uterine cavity [1]. It is one of the most common benign diseases in women, affecting about 10% of all women of reproductive age and 20–50% of infertile women [2]. Noninvasive diagnosis is important, as patients with this condition may go through numerous consultations and examinations, with the time from symptom onset to final diagnosis extending up to 7 years [3].

Superficial endometriosis (also called peritoneal endometriosis) occurs with peritoneal infiltration of less than 5 mm depth; ovarian endometriosis includes superficial ovarian implants and endometriomas; deep endometriosis is characterized by foci of depth greater than 5 mm affecting the retrocervix, paracervix, rectovaginal septum, various portions of the digestive tract (e.g., rectosigmoid), ureter, bladder and can obliterate vesicouterine or retouterine pouchs [4, 5]. Exceptionally, endometriotic implants can be found at more distant sites, including the lung, liver, diaphragm, and operative scars.

Bowel endometriosis occurs in 3%-37% of cases [6], and in 90% of them the rectum or sigmoid colon is involved [7, 8], highlighting the relevance of this particular anatomical region, which can be easily approached and assessed by means of transvaginal sonography (TVS) and magnetic resonance imaging (MRI), the most used noninvasive modalities diagnosis and preoperative mapping of endometriotic lesions [9–15].

In the last two decades, several studies have examined the accuracy of imaging modalities such as TVS and MRI for the diagnosis of deep endometriosis, although just a small subset of them separately addressed the rectosigmoid region [16, 17]. Given the heterogeneity of such studies and their results, this systematic review and meta-analysis were conducted to compare the accuracy of TVS and MRI in the diagnosis of rectosigmoid endometriosis (RE) using only data from studies that compared such modalities within the same set of patients, in order to avoid potential biases compromising external validity when both tests had not been compared within the same population (e.g., reference bias, patient cohort bias, etc). Although a meta-analysis on this subject has been recently published [18], it followed a distinct methodology and selection criteria for included studies, with a smaller number of patients and lower pre-test probability, therefore justifying the addition of a different meta-analysis on this theme to the literature.

## Methods

### Protocol and registration

This systematic review and meta-analysis were conducted in accordance with the PRISMA guidelines [19] (S1 File). The protocol was registered in the PROSPERO international database (www.crd.york.ac.uk/prospero/; no. CRD42017064378).

### Eligibility criteria

The review included cross-sectional studies comparing the accuracy of TVS and MRI for the diagnosis of rectosigmoid endometriosis in patients with suspected deep endometriosis based on clinical history and/or physical examination. Eligible studies applied both modalities to the same patients, followed by surgical and histological confirmation. We imposed no restriction related to details of the technique (e.g., with or without intestinal preparation, introduction of contrast medium by the vaginal and/or rectal route). The main outcome measures were accuracy, sensitivity, specificity, positive and negative predictive values (PPV and NPV), and positive and negative likelihood ratios (LR+ and LR-).

### Literature search

Three independent researchers (APCM, WMB, and RS) searched the MEDLINE (via PubMed), Embase, and Latin American and Caribbean Health Science Literature (LILACS) electronic databases for literature published in Portuguese, English, Spanish, or French through February 2019. The following search strategy was used for the MEDLINE databases (Box 1):

**Box 1. Search strategy used for the MEDLINE databases**

(Endometriosis OR Endometrioses OR Endometrioma OR Endometriomas) AND (Ultrasonography OR Ultrasound OR Ultrasounds OR Sonography OR Echography OR Ultrasonic) AND (Ressonance Magnetic Imaging OR NMR Imaging OR MRI Scan OR MRI Scans OR Imaging, Magnetic Resonance OR MRI) AND (sensitiv*[Title/Abstract] OR sensitivity and specificity[MeSH Terms] OR diagnose[Title/Abstract] OR diagnosed[Title/Abstract] OR diagnoses[Title/Abstract] OR diagnosing[Title/Abstract] OR diagnosis[Title/Abstract] OR diagnostic[Title/Abstract] OR diagnosis[MeSH:noexp] OR diagnostic * [MeSH:noexp] OR diagnosis,differential[MeSH:noexp] OR diagnosis[Subheading:noexp]).

For Embase and LILACS, the search was conducted using the term "endometriosis AND diagnostic." In addition, the reference lists of the selected articles have been manually verified in order to identify potential relevant articles missed in the first step.

### Study selection

**Fig 1:**
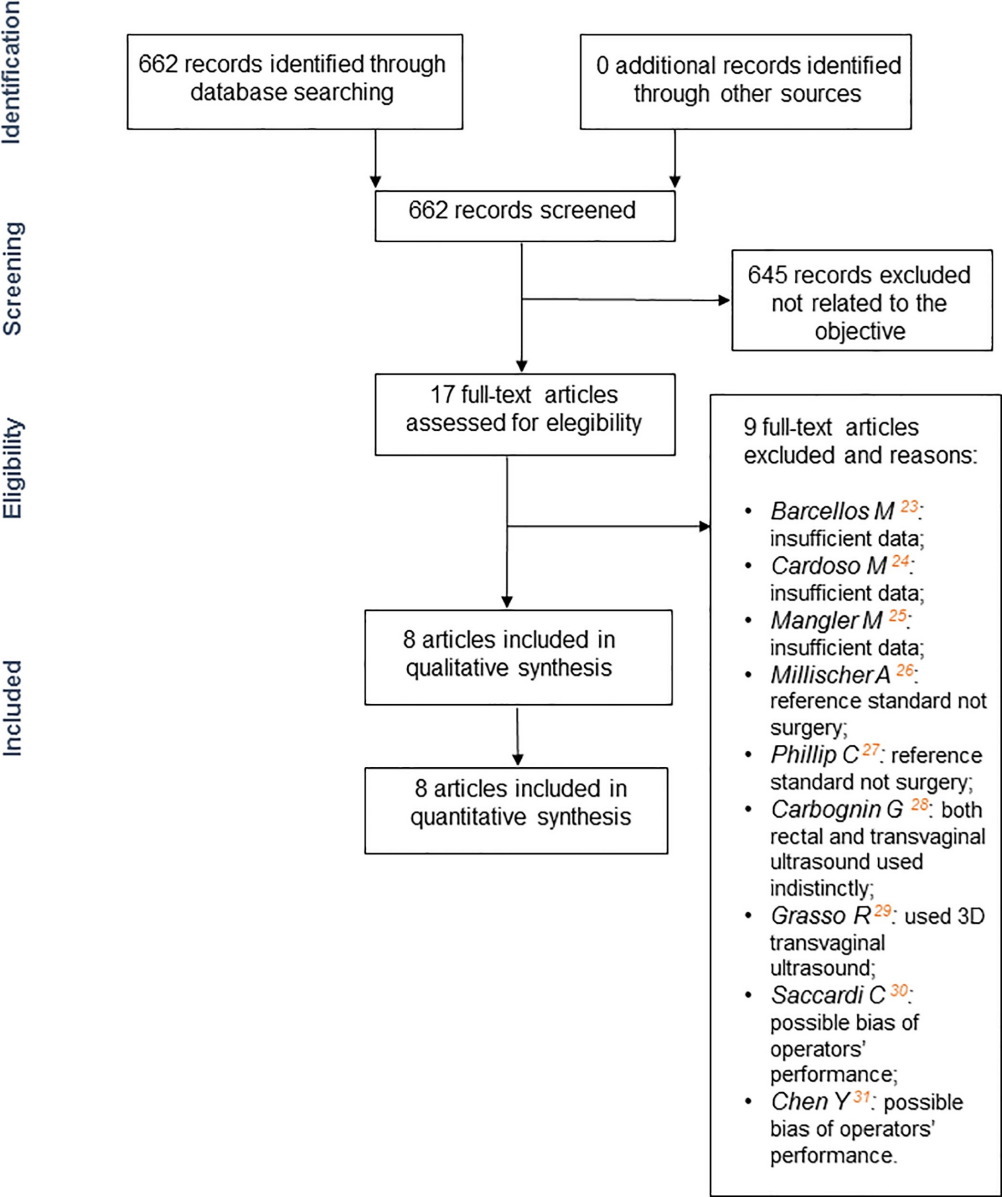
Flowchart of selection process.

The same three researchers (APCM, WMB and RS) independently evaluated the titles and abstracts of identified publications to assess eligibility for inclusion in the review. They then critically evaluated the full texts of original articles. Disagreements were resolved by consensus. The selection process is summarized in Fig 1.

### Data collection

One reviewer (APCM) recorded data from each study using an extraction table for diagnostic studies, and a second reviewer (WMB) checked all extracted data. The following data were extracted: number of patients included, study design, patient characteristics, inclusion and exclusion criteria, TVS and MRI results (number of patients with RE based on the surgical findings and histological confirmation), interval between TVS and MRI examinations, interval between TVS/MRI and gold standard examinations, primary outcomes (including true positive, true negative, false positive and false negative), and secondary outcomes (including accuracy, sensitivity, specificity, PPV, NPV, and LR+/LR- for the diagnosis of RE).

### Risk of bias assessment

To verify the validity of eligible cross-sectional studies, two reviewers (APCM and WMB) independently analyzed the risk of bias using the QUADAS-2 tool [20]. This tool guides assessment of the risk of bias and applicability to the research question in four domains: patient selection, index test, reference (“gold”) standard, and flow (time between test indices and "gold standard" application). The risk of bias was classified as "low," "high," or "unclear". Studies with "low” risk ratings in at least three of the four domains were considered to be of high quality, and those with "high" or "unclear" risk ratings in at least three of the four domains were considered to be of low quality. Studies with all other combinations of ratings were considered to be of moderate quality.

### Analysis

The meta-analysis was performed using the RevMan software (ver. 5.3) [21], obtained from the website of Cochrane Informatics and Department of Knowledge Management. Meta-Disc software (ver. 1.4) [22], available from the Ramón y Cajal University Hospital website (http://www.hrc.es), was used to calculate summary receiving operator characteristic (S-ROC) curves. Both the pretest probability (prevalence) and post-test probability (the chance of RE lesion of deep endometriosis, calculated using meta-analysis data) were also analyzed throughout the studies.

## Results

### Study selection

The initial search generated 662 citations, and 645 articles were excluded after preliminary review of titles and abstracts because they did not address the main objective. Among the 17 articles that fulfilled the eligibility criteria (comparison of the performance of TVS and MRI in the diagnosis of intestinal endometriosis), five were excluded because they did not provide sufficient data (*n* = 3) [23–25] or did not use surgery as the reference standard (*n* = 2) [26, 27]; one study was excluded because vaginal and/or rectal sonography were used indistinctly, precluding the verification of the accuracy of TVS separately [28]; one study was excluded because only tridimensional (rather than conventional) TVS was used [29]; finally, other two articles [30, 31] were excluded because of the possibility of radiologic bias of performers’ experience [32, 33].

Eight studies published between 2007 and 2018 finally met the inclusion criteria, all of them having included surgery and histological analysis as the gold standards [34–41].

### Study characteristics

The eight studies included in the analysis gathered 1132 women who underwent TVS and MRI for suspected endometriosis, based on clinical history (pelvic pain or infertility) and/or physical examination (pain and nodulation on palpation). The main characteristics of the studies are summarized in Table 1.

**Table 1:** Eight final completed studies with their characteristics.

Study	Year	Country	Design of Study	Number of Patients	Gold Standard	Interval	Interval	Inclusion Criteria	Methods	Bowel Preparation
						TVUS x MRI	TVUS/MRI x Reference Standard			
**Abrão**	2007	Brazil	Transversal	104	Surgery Histology	Not Cited	3 months	clinical suspicion of endometriosis	Vaginal examination, TVS and MRI	TVS: yes MRI: no
**Bazot**	2009	France	Transversal	92	Surgery Histology	Not Cited	Not Cited	clinical suspicion of endometriosis	Vaginal and rectal examination, TVS, MRI, RES	TVS: no MRI: no
**Cazalis**	2012	France	Transversal	25	Surgery Histology	Not Cited	Not Cited	clinical suspicion of endometriosis	TAS, TVS and MRI	TVS: no MRI: no
**Saba**	2012	Italy	Transversal	59	Surgery Histology	Not Cited	8 days	clinical suspicion or suspected endometriosis physical examination	TVS and MRI	TVS: yes MRI: no
**Vimercati**	2012	Italy	Transversal	90	Surgery Histology	Not Cited	Not Cited	clinical suspicion or suspected endometriosis image examination	TVS and ColonoMRI	TVS: no MRI: yes
**Maggiore**	2016	Italy	Transversal	286	Surgery Histology	Not Cited	3 months	clinical suspicion of endometriosis	TVS with rectal enema and MRI with rectal enema	TVS: yes MRI: no
**Guerriero**	2018	Spain / Italy	Transversal	159	Surgery Histology	30 days	30 days	clinical suspicion of endometriosis	2D and 3D TVS and MRI	TVS: no MRI: no
**Alborzi**	2018	Iran	Transversal	317	Surgery Histology	Not Cited	Not Cited	clinical suspicion or suspected endometriosis physical examination	TVS, TRS, MRI	TVS: yes MRI: no

TVS: transvaginal sonography; MRI: magnetic resonance imaging; RES: rectal endoscopic sonography; TRS: transrectal sonography

The design, performance, and analysis of results were similar among studies. Examinations in all studies were conducted independently, and the examiners were not aware of the results of physical examination (when appropriate) or other procedures. The protocols used in TVS and MRI in the six selected studies are summarized in Tables 2 and 3, respectively.

**Table 2:** Transvaginal ultrasound protocol.

Study	Transducer (MHz)	Number of Examiners	Bowel Preparation	Bowel Opacification	Vaginal Opacification
**Abrão**	5,0–9,0	1	Yes	N/S	N/S
**Bazot**	5,0–9,0	1	No	N/S	N/S
**Cazalis**	N/S	N/S	No	N/S	N/S
**Saba**	6,5–7,0	1	N/S	N/S	N/S
**Vimercati**	5,0–9,0	N/S	No	No	No
**Maggiore**	N/S	1	Yes	Yes	N/S
**Guerriero**	5,0–9,0	1	N/S	No	No
**Alborzi**	7,5	1	Yes	N/S	N/S

N/S: not stated)

**Table 3:** Magnetic resonance imaging protocol.

Study	Tesla	Bobine	Number of Examiners	Bowel Preparation	Bowel Opacification	Vaginal Opacification	Fast	Antispasmodic	Gadolinium	Resumed Protocol
**Abrão**	1,5	Phased Array	1	No	N/S	Yes	4 hours	Yes	Yes	N/S
**Bazot**	1,5	N/S	1	Yes	N/S	N/S	3 hours	Yes	Yes	T2 ax, T2 sag; T1 GE with and without fatsat
**Cazalis**	N/S	N/S	N/S	Yes	N/S	No	N/S	N/S	Yes	T2 ax, T2 sag, T2 cor; T1 with and without fatsat
**Saba**	1,5	Phased Array	N/S	Yes	N/S	N/S	6 hours	Yes	Yes	T2 ax, T2 sag, T2 cor; T1 ax, T1 sag, T1 cor; T1 fatsat with and without Gd
**Vimercati**	1,5	Phased Array (4 channels)	N/S	Yes	Yes	N/S	N/S	Yes	Yes	T2 ax, T2 sag, T2 cor; T1 ax, T1 sag
**Maggiore**	1,5	Phased Array (8 channels)	1	N/S	Yes	N/S	N/S	No	Yes	T2 ax, T2 sag; T1 fatsat cor and sag; T1 cor; DWI Ax; FIESTA cor
**Guerriero**	1,5	Body Coil	1	N/S	N/S	N/S	3 hours	Yes	Yes	T2 ax, T2 sag, T2 cor; T1 ax, T1 sag, T1 cor; T1 fatsat with and without Gd
**Alborzi**	1,5	Body Coil	1	N/S	N/S	Yes	4 hours	Yes	Yes	T2 ax, T2 sag, T2 cor; T1 ax, T1 sag, T1 cor; T1 fatsat ax and sag with and without Gd

N/S: not stated

### Risk of bias

Overall, the quality of the studies was good (Tables 4 and 5). According to summary QUADAS-2 ratings, seven studies were classified as of high quality and one study was classified as of moderate quality. The risks of bias in the index test and reference standard domains were similar for all studies. In terms of timing and flow, the interval between TVS and MRI examinations was not reported in any study and the interval between the TVS/MRI and reference standard examinations was not reported in three studies [35, 36, 38]; these omissions may have introduced some bias.

**Table 4:** Bias risk according to QUADAS.

			Abrão	Bazot	Cazalis	Saba	Vimecarti	Maggiore	Guerriero	Alborzi
**PATIENT** **SELECTION**	Signaling questions	Was a consecutive or random sample of patients enrolled?	Yes	Yes	No	Yes	Yes	Yes	Yes	Yes
		Was a case-control design avoided?	Yes	Yes	Yes	Yes	Yes	Yes	Yes	Yes
		Did the study avoided inappropriate exclusions?	Yes	Unclear	Unclear	Yes	Yes	Yes	Yes	Yes
	Risk of bias	Could the selection of patients have introduced bias?	Low	Low	Moderate	Low	Low	Low	Low	Low
	Concerns regarding applicability	Are there concerns that the included patients do not match the review question?	Low	Low	Low	Low	Low	Low	Low	Low
**INDEX** **TEST**	Signaling questions	Were the index test results interpreted without knowledge of the results of the reference standard?	Yes	Yes	Yes	Yes	Yes	Yes	Yes	Yes
		If a threshold was used, was it prespecified?	Yes	Yes	Unclear	Yes	Yes	Yes	Yes	Yes
	Risk of bias	Could the conduct or interpretation of the index test have introduced bias?	Low	Low	Low	Low	Low	Low	Low	Low
	Concerns regarding applicability	Are there concerns that the index test, its conduct, or interpretation differ from the review question?	Low	Low	Low	Low	Low	low	Low	Low
**REFERENCE** **STANDARD**	Signaling questions	Is the reference standard likely to correctly classify the target condition?	Yes	Yes	Yes	Yes	Yes	Yes	Yes	Yes
		Were the references standard results interpreted without knowledge of the results of the index test?	Yes	Yes	Yes	Yes	Yes	Yes	Yes	Yes
	Risk of bias	Could the reference standard, its conduct, or its interpretation have introduced bias?	Low	Low	Low	Low	Low	Low	Low	Low
	Concerns regarding applicability	Are there concerns that the target condition as defined by the reference standard does not match the review question?	Low	Low	Low	Low	Low	Low	Low	Low
	Signaling questions	Was there an appropriate interval between index test(s) and reference standard?	Yes	Unclear	Unclear	Yes	Unclear	Yes	Yes	Unclear

**Table 5:** Summary of bias risk according to QUADAS-2 (L: low risk, H: high risk, M: moderate risk).

Study	Patient Selection	Index Test	Reference Standard	Flow and Timing	Study Quality
**Abrão**	L	L	L	L	High
**Bazot**	L	L	L	M	High
**Cazalis**	H	L	L	M	Moderate
**Saba**	L	L	L	L	High
**Vimercati**	L	L	L	M	High
**Maggiore**	L	L	L	L	High
**Guerriero**	L	L	L	L	High
**Alborzi**	L	L	L	M	High

### Results of quantitative analysis

Table 6 lists the results of quantitative analysis. Prevalence (pretest probability) values ranged from 16.4 to 76%. Sensitivity values ranged from 73.3% to 98.1% for TVS and from 73.3% to 100.0% for MRI. Specificity values ranged from 66.7% to 100.0% for TVS and from 50.0% to 100.0% for MRI.

**Table 6:** Diagnostic measures of transvaginal sonography and magnetic resonance imaging in the diagnosis of deep rectosigmoid endometriosis.

Study	Abrão	Bazot	Cazalis	Saba	Vimercati	Maggiore	Guerriero	Alborzi
**Prevalence**	51,9	68,5	76	50,8	20	52,8	42	16,4
**Sensitivity**								
*TVS*	98.1	93.6	73.7	73.3	77.8	92.7	84.8	88.4
*MRI*	83.3	87.3	89.5	73.3	100.0	95.3	92.4	76.9
**Specificity**								
*TVS*	100.0	100.0	66.7	86.2	94.4	97.0	87.1	98.8
*MRI*	98.0	93.1	50.0	89.6	100.0	97.7	94.6	96.7
**PPV**								
*TVS*	100.0	100.0	87.5	84.6	77.8	97.2	82.4	93.9
*MRI*	97.8	96.5	85.0	88.0	100.0	97.9	92.4	81.63
**NPV**								
*TVS*	98.0	87.9	44.4	75.7	94.4	92.2	89.0	97.8
*MRI*	84.4	77.1	60.0	76.5	100.0	94.9	94.6	95.5
**LR+**								
*TVS*			2.21	5.32	14.0	31.29	6.57	78.14
*MRI*	41.67	12.66	1.79	7.09		42.91	17.2	22.65
**LR-**								
*TVS*	0.02	0.06	0.39	0.31	0.24	0.08	0.174	0.12
*MRI*	0.17	0.14	0.21	0.30	0.00	0.05	0.08	0.24
**Accuracy**								
*TVS*	99.0	95.6	72.0	79.6	91.1	94.7	86.1	97.2
*MRI*	90.3	89.1	80.0	81.3	100.0	96.5	93.7	93.4

TVS: transvaginal sonography; MRI: magnetic resonance imaging

### Comparative accuracy of TVS and MRI

**Fig 2:**
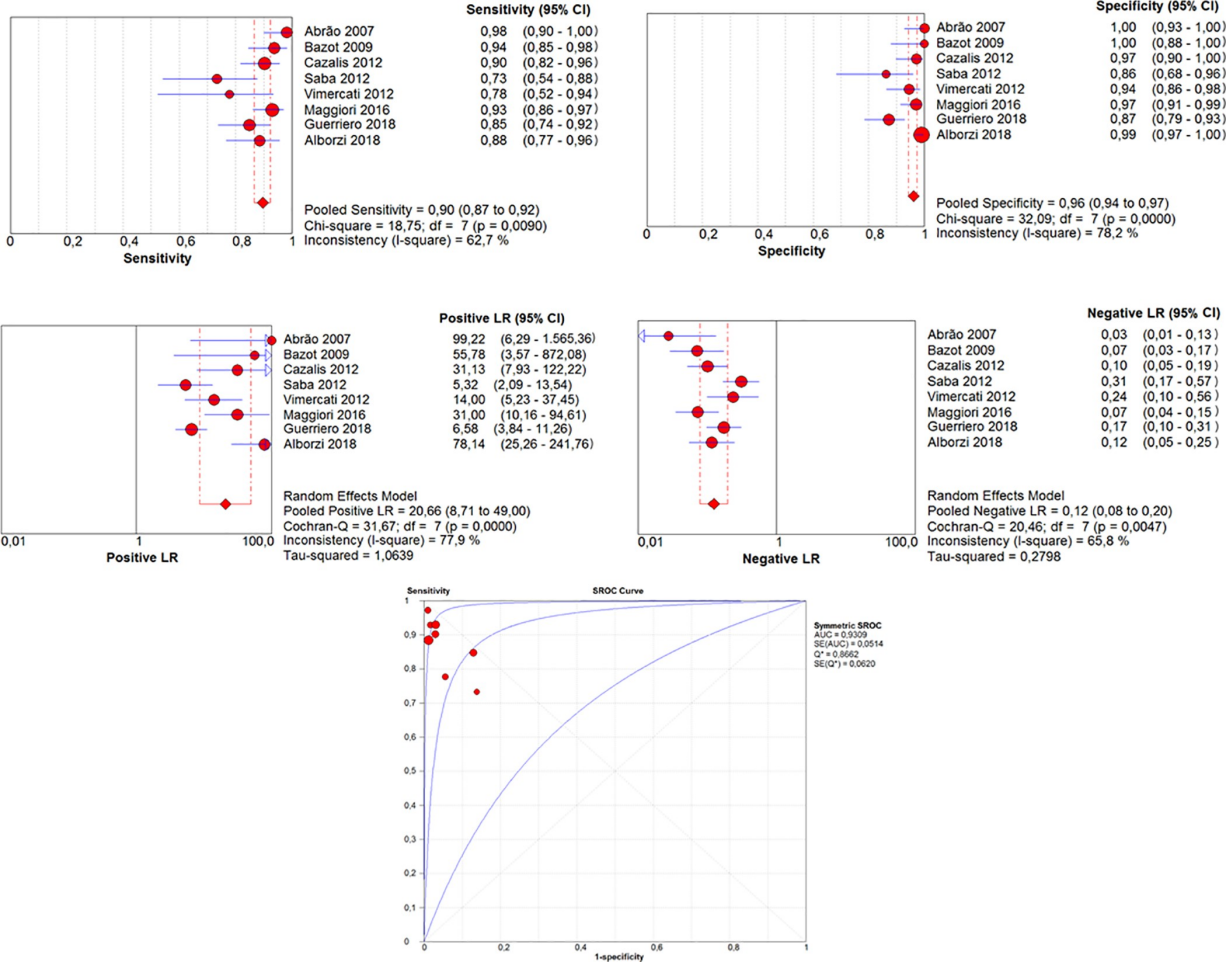
Results of TVS for the diagnosis of rectosigmoid endometriosis.

**Fig 3:**
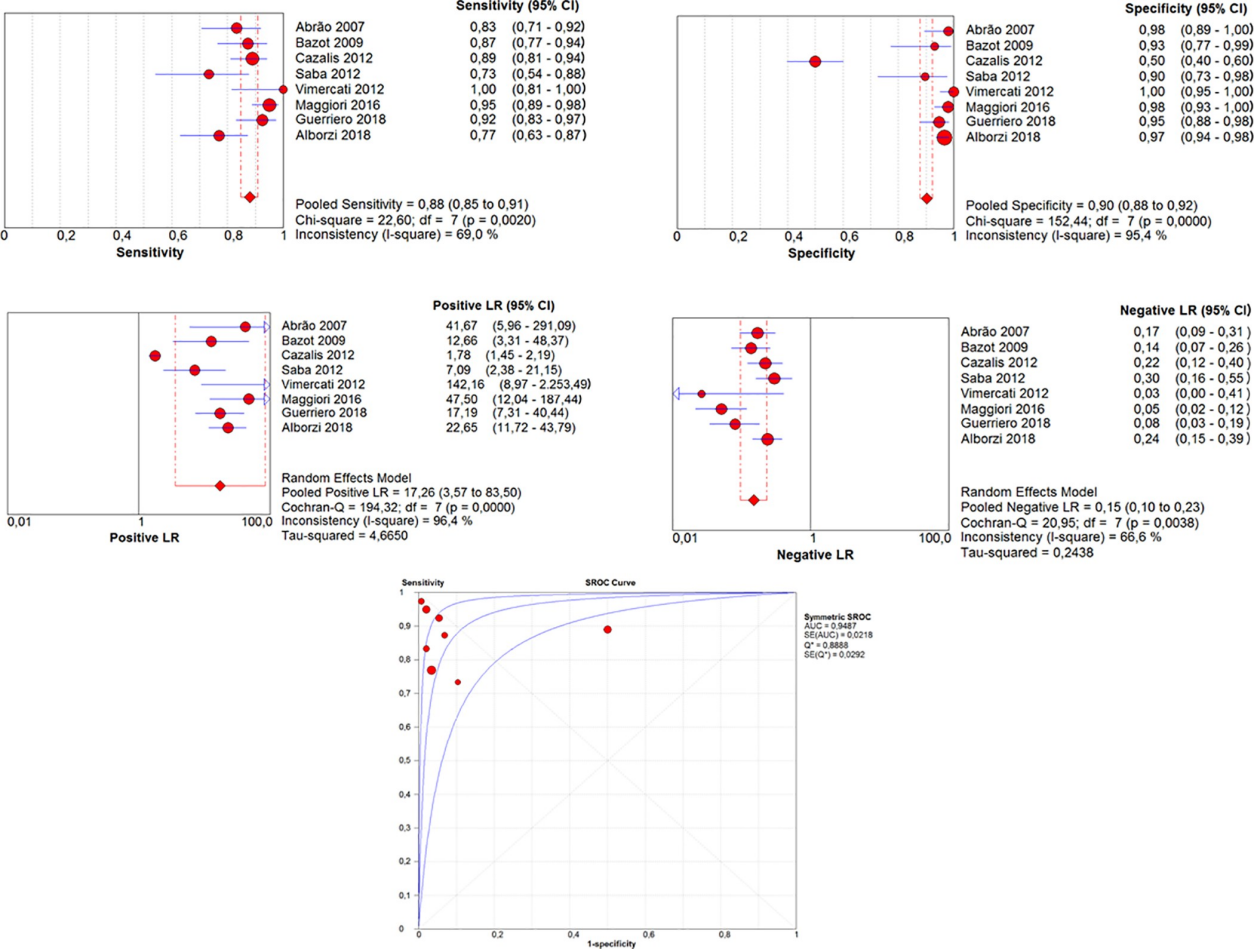
Results of MRI for the diagnosis of rectosigmoid endometriosis.

Sensitivity, specificity, LR+, and LR- values are depicted as forest plots and S-ROC curves (Figs 2 and 3). The areas under the S-ROC curves (AUC) reflected similar accuracy of MRI (AUC = 0.948) and VS (AUC = 0.930) in the diagnosis of RE (*P* = 0.13). Post-test probability values (for positive test results) were 93.9% for TVS, 94.8% for MRI, and 99.6% for the combination of both examinations.

## Discussion

Interest in the noninvasive diagnosis of deep endometriosis using laboratory tests (serological markers) or imaging examinations is increasing [42]. In recent decades, TVS and MRI have been shown to be more accurate than other imaging methods (i.e., transrectal sonography, barium enema, computed tomography colonography) in the detection of RE; they are also less invasive and do not require sedation. Few studies, however, have compared the accuracy of TVS and MRI in the diagnosis of RE in the same set of patients. Comparison of these methods by applying them inhomogeneously (just one or another) in different populations in order to subsequently pooling results and comparing accuracy values across studies is methodologically problematic because each study may have inherent biases (e.g., reference bias, patient cohort bias, etc) that, despite not limiting their internal validity, may compromise their external validity. Moreover, some of these studies were performed with small sample sizes, which limit their statistical power and makes it a meta-analytic approach desirable.

This meta-analysis included eight studies with a total of 1132 patients who underwent TVS and MRI, enabling comparison of the performance of these modalities in the same population. It showed that MRI and TVS have similar performance in the diagnosis of rectosigmoid endometriosis. Moreover, the post-test probability findings for TVS and MRI did not differ. The study findings suggest that the combined use of TVS and MRI is reasonable, as the chance of noninvasively and accurately diagnosing RE rises to practically 100% when both examinations yield positive results.

Three recent systematic reviews [18, 42, 43] have involved the comparison of noninvasive diagnostic tests for endometriosis, demonstrating the interest of the scientific community in identifying accurate modalities for this purpose. Nisenblat et al. [42], despite presenting separate mean estimates for each imaging modality (TVS, 14 studies, 15 data sets, 1616 participants, sensitivity of 0.90 and specificity of 0.96; MRI, six studies, seven data sets, 612 participants, sensitivity of 0.92 and specificity of 0.96) have not meta-analyzed studies directly comparing these modalities.

Guerriero et al. [18], compared TVS and MRI findings from studies employing the same population. For MRI detection of RE, they found nearly similar sensitivity and specificity values in comparison to us (of 0.85 and 0.95, respectively, *versus* the values of 0.88 and 0.90 found in our study). For TVS, these values were of 0.85 and 0.96, respectively, against 0.90 and 0.96 found in our study. Methodological differences between the two meta-analyses, however, must be taken into account, especially in terms of study selection. Given the recent date of our meta-analysis, for example, we included three new additional studies, by Maggiore et al. [39], Guerriero et al. [40] and Alborzi et al. [41]. Maggiore et al. [39] and Alborzi et al. [41] published the two largest series of patients to date (n = 286 and 317, respectively). Therefore, our meta-analysis encompassed a total of 1132 patients in a 12-year timeframe (from 2007–2018), which means 62.6% more than Guerriero et al.’s study [18]. Finally, despite having found no significant statistical difference between such methods in terms of pooled sensitivity and specificity, these authors did not perform comparisons of the AUC values for both modalities, nor estimated the impact of combining such modalities on the positive post-test probabilities of a RE diagnosis, as did we.

Finally, Bazot et al. [43] provided an overview of published reviews and discussed the imaging protocols, the definition of endometriosis *per se*, and the need for laparoscopic and histological confirmation in the diagnosis of the disease.

The studies included in this review and meta-analysis have some concerns that should be addressed. The first issue is bowel preparation, which has been shown to increase the accuracy of intestinal lesion detection [44]. No study included in this analysis involved intestinal preparation for both TVS and MRI; this procedure was performed for one examination in some studies [34, 37–39] and for neither examination in others [35, 36]. Even without standard bowel preparation, however, the rate of rectosigmoid endometriosis detection was quite high in these studies. The second concern regards the intestinal endometriosis represented only by rectosigmoid disease, preventing that we could extend the scope of such meta-analysis to other intestinal locations beyond the rectosigmoid. Other locations, especially those in the right iliac fossa (cecum, appendix, and ileum) were not mentioned in any study. In all studies, only the rectosigmoid colon was investigated, which represents a limitation in the evaluation of all intestinal endometriotic lesions. The third issue is that no study involved the comparison of lesion characteristics, such as size (along three axes), maximum depth of the affected layer, and circumference. These characteristics have great impact on surgical planning, including the selection of intestinal resection type (linear, discoidal, or segmental). The last issue regards to the prevalence of rectosigmoid endometriosis, which ranged from 16.4 to 76%. Selection bias may have affected the diagnostic performance of the imaging examinations, as reported by Guerriero et al. [18].

Regarding the choice between MRI and US for diagnosing rectosigmoid endometriosis, some aspects should be considered. US is a safe, low cost and widespread technique, with similar diagnostic accuracy and post-test probabilities in comparison to MRI in terms of available published data. However, US is also operator-dependent, and errors in technique, equipment quality, and limitations of operator’s experience are all factors that may lead to misinterpretations and misdiagnoses [45]. MRI, on the other hand, despite its higher costs, has useful advantages in the context of deep pelvic endometriosis, such as the capacity of obtaining multiplanar sequences with distinct relaxation times, allowing an unmatched ability to differentiate normal from diseased tissues [45]; moreover, the standardized obtained images by the MRI scanner can be interpreted remotely by an expert (rising the accuracy), while with US the process of obtaining the diagnostic images depends inherently on the ability and experience of the operator. Therefore, the rationale behind the choice of these methods should take into account a range of variables, such as local characteristics of the institution, availability of each method, costs, available budget, radiologists’ experience with each method, etc. In an ideal scenario (an institution with high expertise in imaging of endometriosis), we think that TVS could be used as an initial method, while MRI could be reserved in doubtful cases or to rise the post-test probabilites to near 100%. Moreover, MRI could be used in the preoperative planning of patients selected for surgical treatment.

## Conclusion

The noninvasive diagnosis of RE can be made based on MRI and TVS with good sensitivity and specificity. The review and meta-analysis revealed that both methods have high and similar values of diagnostic accuracy and positive post-test probabilities. The state of art in the diagnostic imaging management of RE should combine the two methods. Both examinations can be performed on the same day, requiring a single bowel preparation, which we believe is important to increase the detection rate of small lesions.

## Supporting information

S1 File PRISMA guidelines checklist. (DOC)
