# Correction: Accuracy of transvaginal sonography versus magnetic resonance imaging in the diagnosis of rectosigmoid endometriosis: Systematic review and meta-analysis

**DOI:** 10.1371/journal.pone.0221499

**Published:** 2019-08-16

**Authors:** Ana Paula Carvalhal Moura, Helizabet Salomão Abdalla Ayroza Ribeiro, Wanderley Marques Bernardo, Ricardo Simões, Ulysses S. Torres, Giuseppe D’Ippolito, Marc Bazot, Paulo Augusto Ayrosa Galvão Ribeiro

In the Results section of the Abstract, there is an error in the second sentence. The correct sentence is: The pooled sensitivity, specificity, LR+, and LR- values of MRI for RE were 88% (95% CI, 85–91%), 90% (95% CI, 88–92%), 17.26 (95% CI, 3.57–83.50), and 0.15 (95% CI, 0.10–0.23); values of TVS were 90% [95% CI, 87–92%], 96% (95% CI, 94–97%), 20.66 (95% CI, 8.71–49.00) and 0.12 (95% CI, 0.08–0.20), respectively.

## References

[1] Moura APC, Ribeiro HSAA, Bernardo WM, Simões R, Torres US, D’Ippolito G, et al (2019) Accuracy of transvaginal sonography versus magnetic resonance imaging in the diagnosis of rectosigmoid endometriosis: Systematic review and meta-analysis. PLoS ONE 14(4): e0214842 10.1371/journal.pone.0214842 30964888 PMC6456198

